# Antibacterial activity of *Nymphaea nouchali* (Burm. f) flower

**DOI:** 10.1186/1476-0711-12-27

**Published:** 2013-10-07

**Authors:** Biplab Kumar Dash, Monokesh Kumer Sen, Khasrul Alam, Kamal Hossain, Rezuanul Islam, Nilufa Akhter Banu, Shahedur Rahman, Abu Hena Mostofa Jamal

**Affiliations:** 1 Department of Biotechnology and Genetic Engineering, Islamic University, Kushtia 7003, Bangladesh; 2 Department of Genetic Engineering and Biotechnology, Jessore Science and Technology University, Jessore 7408, Bangladesh

**Keywords:** *Nymphaea nouchali* flower, Antibacterial activity, Disc diffusion assay, Nalidixic acid

## Abstract

**Background:**

The present work aimed to find out the antibacterial activity of *Nymphaea nouchali* flower on human and plant pathogenic bacteria.

**Methods:**

Antibacterial potency of methanol, acetone, ethyl acetate and petroleum spirit extracts of *Nymphaea nouchali* flower has been tested against four human pathogenic bacteria *Bacillus subtilis* (FO 3026) *Escherichia coli* (IFO 3007), *Klebsiella pneumonia* (ATTC 10031) and *Sarcina lutea* (IFO 3232) and one plant pathogenic bacterium *Xanthomonas campestris* (IAM 1671) by disc diffusion assay. Zone of inhibition produced by different extracts against the test bacteria was measured and compared with standard antibiotic disc.

**Results:**

Methanol extract possessed better antibacterial activity against two pathogenic bacteria, *B*. *subtilis* (FO 3026) and *S*. *lutea* (IFO 3232) than commercial antibiotic nalidixic acid. Acetone extract showed moderate sensitivity whereas *B*. *subtilis* (FO 3026), *S*. *lutea* (IFO 3232) and *X*. *campestris* (IAM 1671) showed resistance to ethyl acetate and petroleum spirit extracts. The minimum inhibitory concentrations of various extracts were ranged between 128–2048 μgml^-1^.

**Conclusions:**

*Nymphaea nouchali* flower could be a potential candidate for future development of novel broad spectrum antibacterial herbal formulation.

## Introduction

The emergence of multiple antibiotic resistance pathogenic bacterial strains is a major medical problem worldwide and poses a big threat to human society [1,2]. Moreover almost all of the antibiotics have side effects including hypersensitivity, immune-suppression and allergic reactions, and they are expensive too [3]. These circumstances make it essential to search new and more potent antimicrobial compounds to combat these pathogens. Herbal treatment is one possible way to treat disease caused by multidrug resistant bacteria [1,4,5] which is the basis for this study.

*Nymphaea nouchali* (Burm. f) (Water lily in English and Shapla in Bangla) is the national flower of Bangladesh. It is an aquatic rooting herb belongs to the family Nympheaceae generally found in lakes and ponds throughout the country [6]. Various secondary metabolites like sterols (nymphayol, isolated from flower), alkaloids, saponins, tannins, and flavonoids has been isolated from this plant [7] and these metabolites may be responsible for antibacterial activities. It has been reported to use in treatment of diabetes, tumor, inflammation, liver and urinary disorders, menstruation problems, indigestion and also used as food by the local people [7-11]. There are many literatures reporting the ethno-medicinal values of *N*. *nouchali*, but there is little scientific proof for further using this plant commercially or in a more effective form. Therefore, an attempt was made to evaluate the antibacterial potential of *N*. *nouchali* flower extracts against human and plant pathogenic bacteria.

## Materials and methods

### Plant material

Healthy, disease free *N*. *nouchali* flower collected from different ponds of Kushtia and Jhenidah region, Bangladesh during the month of July, 2012. This plant was then botanically identified by Bushra Khan, Principal Scientific Officer, National Herbarium, Mirpur, Dhaka 1216, Bangladesh. A voucher (DACB 38572) has been deposited in National Herbarium, Mirpur, Dhaka 1216, Bangladesh.

### Preparation of extracts

Fresh flowers were cleaned with deionized water and dried in shade for two to four weeks. After drying, the flowers were pulverized into fine powder by a grinding machine and stored in airtight container. This powder was used to prepare methanol, ethyl acetate, acetone and petroleum spirit extracts. These crude extracts were then filtered and concentrated by using a rotary evaporator. After that, the extracts were diluted to 4096, 2048, 1024, 512, 256, 128, 64, 32, 16, 8, 4, 2 and 1 μgml^-1^. All the extracts were stored in refrigerator at 4°C in sterile container for further use [12].

### Test bacteria

Pure culture of *Bacillus subtilis* FO 3026, *Sarcina lutea* IFO 3232, *Xanthomonas campestris* IAM 1671, *Escherichia coli* IFO 3007, *Klebsiella pneumonia* ATTC 10031 obtained from the Microbiology Laboratory of Department of Biotechnology and Genetic Engineering, Islamic University, Kushtia, Bangladesh and were used in this study (10^6^ CFUml^-1^).

### Antibacterial bioassay

Disc diffusion method was used for *in vitro* antibacterial activity assesment [13]. Filter paper discs (6 mm diameter) of various extracts (methanol, ethyl acetate, acetone and petroleum spirit) were prepared by impregnating blank sterile paper discs to the respective extracts with different concentrations (4096, 2048, 1024, 512, 256, 128, 64, 32, 16, 8, 4, 2 and 1 μgml^-1^). These paper discs were placed on nutrient agar inoculated with the test bacteria and incubated at 37°C for 24 h. Nalidixic acid (30 μg/disc) (Invitrogen, USA) were used as positive control and blank discs (impregnated with solvents followed by evaporation) were used as negative control. After incubation the culture plates were examined and the zones of inhibition were measured in millimeter scale [14].

### Statistical analysis

Each experiment was run in triplicate, and mean values were calculated with SD (standard deviation). SPSS version 11.0 was used for the data analysis.

## Results

### Antibacterial activities

**Figure 1:**
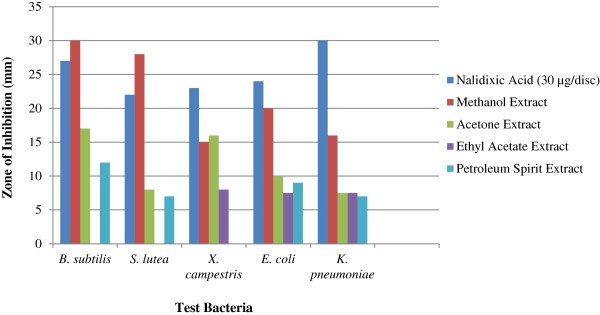
**Antibacterial activity of different extracts of *N*. *nouchali *flower (4096 μgml**^**-1**^**).**

The antibacterial activity of different extracts of *N*. *nouchali* flower is shown in Figure 1 in a comparative way with standard antibiotic disc- nalidixic acid (30 μg/disc). The present study revealed that, methanol extract possessed the highest zone of inhibition of 30 ± 0.18 mm against *B*. *subtilis* followed by 27 ± 0.28 mm and 20 ± 0.60 mm against *S*. *lutea* and *E*. *coli*, respectively. Acetone extract produced satisfactory sensitivity of 17 ± 0.23 mm and 16 ± 0.62 mm against *B*. *subtilis* and *X*. *campestris*, respectively. Ethyl acetate and petroleum spirit extracts showed relatively poor antibacterial activity. All the test extracts were somehow effective against both *K*. *pneumoniae* and *E*. *coli* whereas both *B*. *subtilis* and *S*. *lutea* were resistant by ethyl acetate extract. Petroleum sprit extract failed to inhibit the growth of *X*. *campestris*. Positive control produced significant zones of inhibition against all the test bacteria while no zone was formed by negative controls.

### Minimum inhibitory concentration (MIC)

**Figure 2:**
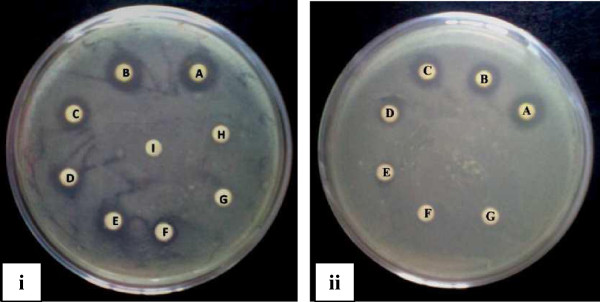
**Methanol extract of *N. nouchali *flower produce zones of inhibition at different concentrations against (i) *B. subtilis *and (ii) *S. lutea.***

The result of MIC assay is shown in Table 1. MIC values of various extracts were found between 128–2048 μgml^-1^. The best MIC value was 128 μgml^-1^ against *B*. *subtilis* by methanol extract producing 4.0 ± 0.00 mm zone of inhibition (Figure 2).

**Table 1:** **MIC values of various extracts of *N. nouchali *flower**

**Bacterial strains**	**Methanol extract**		**Acetone extract**		**Ethyl acetate extract**		**Petroleum spirit extract**	
	**Concentration (μgml**^**-1**^**)**	**DIZ (mm)**	**Concentration (μgml**^**-1**^**)**	**DIZ (mm)**	**Concentration (μgml**^**-1**^**)**	**DIZ (mm)**	**Concentration (μgml**^**-1**^**)**	**DIZ (mm)**
*B. subtilis*	128	4.0 ± 0.00	256	3.5 ± 0.04	-	-	516	2.5 ± 0.01
*S*. *lutea*	256	3.2 ± 0.00	1024	1.9 ± 0.03	-	-	1024	1.9 ± 0.03
*X*. *campestris*	1024	2.0 ± 0.00	512	2.8 ± 0.05	1024	1.8 ± 0.13	-	-
*E*. *coli*	512	3.5 ± 0.03	1024	2.3 ± 0.11	2048	2.0 ± 0.05	2048	1.2 ± 0.07
*K*. *pneumoniae*	1024	3.3 ± 0.00	2048	1.5 ± 0.07	1024	1.8 ± 0.00	2048	1.0 ± 0.00

DIZ = Diameter of Inhibition Zone.

Concentration of disc A, B, C, D, E, F, G, H and I are 4096, 2048, 1024, 512, 256, 128, 64, 32 and 16 μgml^-1^ respectively.

## Discussion

Infectious diseases caused by bacteria, fungi, viruses and parasites are a major threat to public health in developing countries due to unavailability and high cost of medicines [15,16]. This enabled us to evaluate *N*. *nouchali* flowers for its antimicrobial activity.

In this study methanol extract of *N*. *nouchali* flower showed better activity against both *B*. *subtilis* and *S*. *lutea* than nalidixic acid, a broad spectrum synthetic quinolone antibiotic, and it is mentionable that the antibacterial efficiency is 11% greater against *B*. *subtilis* as well as 27% greater against *S*. *lutea* than the antibiotic. The ability of plant extract to kill or inhibit pathogenic bacterial growth with great efficiency indicates the presence of some active compounds which have antibacterial activity. *E*. *coli* and *K*. *pneumoniae*, other two human pathogens also showed satisfactory sensitivity to methanol extract. *B*. *subtilis* was the most susceptible bacteria to all except the ethyl acetate extract. *X*. *campestris*, a pathogen for cabbage and cauliflower inhibited significantly by both methanol and acetone extracts; this suggests the effectiveness of this plant against plant pathogen. The MIC study also suggest that methanol and acetone extracts of *N*. *nouchali* flower could become an alternative to synthetic bactericides for using in pharmaceutical industry to control some pathogenic bacteria.

The antibacterial activity is believed to be due to the presence of secondary metabolites like alkaloids, tannins, steroids, phenol, saponins, flavonoids compounds, which are previously reported for their antimicrobial property [9-11]. It is therefore conceivable that these extract could be used against infections caused by these inhibited bacteria and the results showed a good correlation between the reported use of *N*. *nouchali* in traditional medicine against infectious diseases and in *in vitro* effectiveness. This study may not be adequate to suggest potential antibiotic agent considering the MIC value, and the zone of inhibition could be affected by the solubility and rate of diffusion in agar medium or its volatilization which could affect the results. However, this approach could be considered as preliminary step to find out promising candidates [1]. It is essential to do the quantitative analysis with the help of a chromatographer in gas phase and identify the metabolites responsible for antibacterial activity.

## Conclusions

The outcome of present study suggests that some of the *N*. *nouchali* flower extracts possess compounds with high antibacterial properties that can be used as antibacterial agents in designing and developing new drugs. Further purification and characterization of the active compounds will provide a better understanding of the antibacterial mechanism.

## Competing interests

The authors declare that they have no competing interests.

## Authors’ contributions

BKD, MKS, KS and KH conducted laboratory analysis and drafted the manuscript. RI, NAB, SR and AHMJ participated in the design of the study and supervised the work. All authors were involved in the interpretation of the data and approved the final manuscript.
